# β-Hydroxy sulfides and their syntheses

**DOI:** 10.3762/bjoc.14.143

**Published:** 2018-07-05

**Authors:** Mokgethwa Bruce Marakalala, Edwin M Mmutlane, Henok H Kinfe

**Affiliations:** 1 Department of Chemistry, University of Johannesburg, PO Box 524, Auckland Park 2006, South Africa

**Keywords:** alkene thiofunctionalization, epoxide thiolysis, β**-**hydroxy sulfides, sulfur-containing natural products

## Abstract

Sulfur-containing natural products are ubiquitous in nature, their most abundant source being marine organisms since sulfur, in the form of the sulfate ion, is the second most abundant anion in sea water after chloride. As part of natural products, sulfur can appear in a multitude of combinations and oxidation states: thiol, sulfide (acyclic or heterocyclic), disulfide, sulfoxide, sulfonate, thioaminal, hemithioacetal, various thioesters, thiocarbamate and isothiocyanate. This review article focuses on β-hydroxy sulfides and analogs; their presence in natural products, general protocols for their synthesis, and examples of their application in target oriented synthesis.

## Review

### 1. Introduction

**Figure 1:**
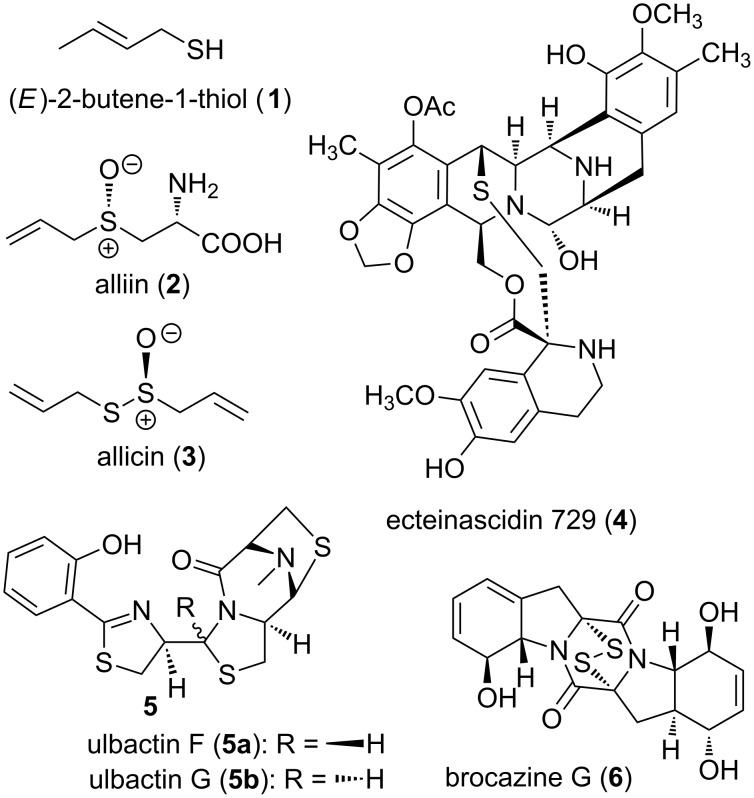
Some sulfur-containing natural products.

Organosulfur compounds are widely distributed in nature, with marine organisms being the richest sources of these, since sulfur, as the sulfate ion, is the second most abundant anion in sea water after chloride [1]. Marine natural products in general, have for years been the subject of annual reviews [2]. Those marine natural products containing sulfur have also received their own, separate attention [1,3]. As can be gleaned from the selected examples in Figure 1, naturally occurring sulfur-containing organic compounds are, like all natural products/secondary metabolites, staggeringly diverse with respect to their natural source and their level of structural complexity. As part of their molecular architecture, sulfur can appear in the form of various functional groups and oxidation states: thiol, sulfide (acyclic or heterocyclic), disulfide, sulfoxide, sulfonate, thioaminal, hemithioacetal, various thioesters, thiocarbamate and isothiocyanate [3].

The three simplest sulfur-containing natural products are perhaps, (*E*)-2-butene-1-thiol (**1**), the principal ingredient of the repulsively malodorous skunk oil [4], alliin (**2**), the precursor to 1-propenesulfinic acid which rearranges to the sulfine (*Z*)-propanethial-*S*-oxide, the lachrymatory factor of onions, (*Allium cepa L*), and allicin (**3**), the thiosulfinate with antibiotic properties from garlic (*Allium sativum L*.) [5]. On the other molecular architectural extreme are: the ecteinascidins, antitumor tetrahydroisoquinoline alkaloids from the colonial Ascidian *Ecteinascidia turbinata* as exemplified by ecteinascidin 729 (**4**) [6]; ulbactins F (**5a**) and G (**5b**), two polycyclic thiazoline congeners isolated from a culture extract of a sponge derived *Brevibacillus* sp. collected off the coast of Japan [7]; and brocazine G (**6**), a bisthiodiketopiperazine which displayed potent cytotoxicity to sensitive and cisplatin resistant human tumor cell lines (HTCLs) and strong activity against *S. aureus* [8]*.* Further examples illustrating the structural diversity and fascinating biosynthesis of sulfur-containing secondary metabolites can be found in a recent review by Hertweck and co-workers [9].

### 2. β-Hydroxy sulfides

**Figure 2:**
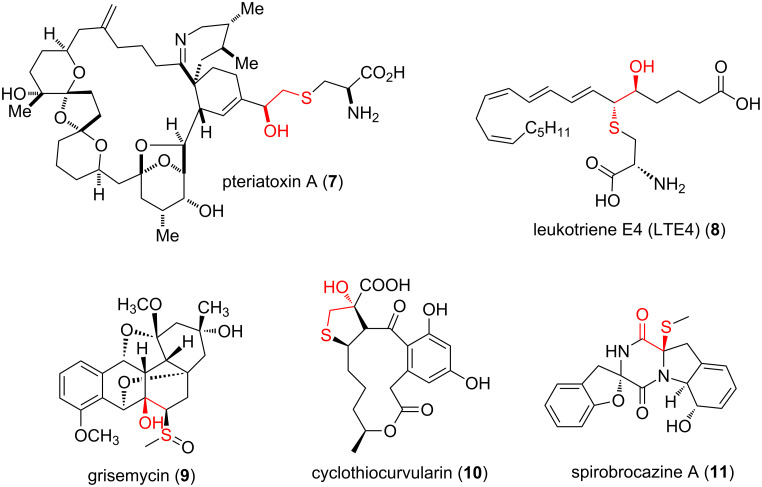
Some natural products incorporating β-hydroxy sulfide moieties.

β-Hydroxy sulfides, often in disguised form, comprise a significant segment of sulfur-containing natural products, with a few examples shown in Figure 2. The pteriatoxins such as pteriatoxin A (**7**), were isolated from a Japanese shellfish by Uemura and co-workers in 2001 and proved to be extremely potent neurotoxins in mice [10]. Leukotrienes, which are a family of eicosanoids that are produced in leukocytes, also contain β-hydroxy sulfide moieties, as exemplified by leukotriene E4 (**8**) isolated from the mast cells and extensively studied for allergy and asthma [11]. Another example is grisemycin (**9**) from *Streptomyces griseus* M268, which contains an unusual ether-bridged system and a methylsulfinyl substituent [12]. Cyclothiocurvularin (**10**), isolated from *Penicillum* sp. [13], and spirobrocazine A (**11**), isolated from mangrove *Penicillum* sp. [8] are further examples.

**Figure 3:**
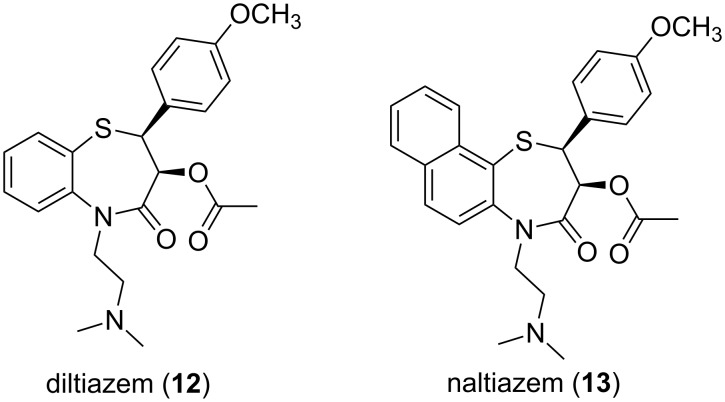
Some synthetic β-hydroxy sulfides of clinical value.

β*-*Hydroxy sulfides are also important functional units found in a number of biologically important synthetic compounds. Examples are diltiazem (**12**) and naltiazem (**13**, Figure 3), calcium channel blockers used in the treatment of hypertension, angina pectoris and some types of cardiac arrhythmia [14].

### 3. Synthetic methods to β-hydroxy sulfides

β-Hydroxy sulfides are important structural units found in a number of biologically important compounds. Furthermore, they have been used as key intermediates in the synthesis of pharmaceuticals and various chemical entities such as allylic alcohols, benzoxathiepines, benzotiazepines, α-thioketones, α-substituted α,β-unsaturated enones, and β-hydroxysulfoxides [15]. Accordingly, a number of methodologies have been reported for their synthesis. Regioselective epoxide ring opening and 1,2-difunctionalization of alkenes are the commonly employed routes in the synthesis of such compounds. Both strategies are discussed below.

#### 3.1 Synthesis of β-hydroxy sulfides via regioselective ring opening of epoxides

The considerable ring strain present in epoxides makes them highly susceptible to nucleophilic attack and makes them versatile starting materials for the synthesis of various derivatives [16–18]. A number of protocols have been reported to effect their selective ring opening using thiols or disulfides as nucleophiles to obtain β-hydroxy sulfides.

**3.1.1 Thiols as nucleophiles.** Thiols, in the presence of a wide variety of catalysts, yield sulfides upon opening of epoxides under a variety of reaction conditions and these have been comprehensively reviewed [19]. More catalysts and reaction conditions, novel or modern variations of the old, keep getting disclosed and are surveyed below.

**Scheme 1:**
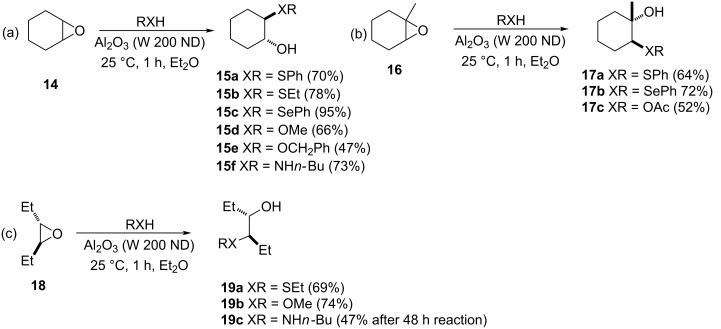
Alumina-mediated synthesis of β*-*hydroxy sulfides, ethers, amines and selenides from epoxides.

**3.1.1.1 Alumina catalysis.** With respect to heterogeneous catalysts, Posner and Rogers reported that inactivated Woelm-200 chromatographic alumina catalyzes a regioselective and stereospecific (in favor of the *trans-*isomer) opening of a wide variety of epoxides by thiols to give various β-hydroxy sulfides in good yields (Scheme 1) [20]. Both aryl and alkylthiols were found to be capable of opening the epoxide ring under the reaction conditions, and with respect to the epoxides, cycloalkane, alkene and 1-substituted alkene oxides were all amenable to the reaction. Besides the ability of the catalysts to promote the epoxide ring opening with sulfur nucleophiles at room temperature, the attractiveness of the promoter was its capability to effect opening of the epoxide ring with alcohols, selenols and amines to provide the corresponding β-hydroxy analogs.

**Scheme 2:**
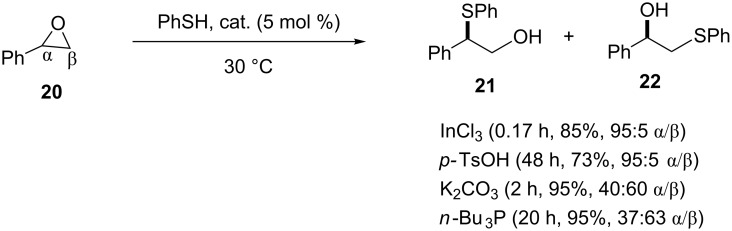
β-Hydroxy sulfide syntheses by ring opening of epoxides under different Lewis and Brønsted acid and base catalysts.

**3.1.1.2 Brønsted and Lewis acid and base catalysis.** Pizzo and co-workers conducted a comparative study on the catalytic activity of Lewis and Brønsted acids as well as bases for the thiolysis of epoxides using InCl_3_, *p-*TsOH, *n-*Bu_3_P and K_2_CO_3_ as representative examples, respectively. Although all of them effected the thiolysis of alkyl and aryl epoxides under solvent-free conditions with 5 mol % of catalyst loading, the activity was highly dependent of the nature of the acid or base catalyst employed. The Lewis acid InCl_3_ and the Brønsted base K_2_CO_3_ provided superior yields and reaction rates in comparison to their corresponding analogs (Brønsted acid *p*-TsOH and Lewis base *n*-Bu_3_P, respectively) as shown in Scheme 2 [21]. In comparison to the reaction carried out under the same reaction conditions but in aqueous or organic media, it was found that the solvent-free reactions provided the β-hydroxy sulfides in reasonable yields over shorter reaction times and at low catalyst loading. The regioselectivity of the reactions was strongly dependent on the acidity or basicity of the reaction conditions. Under acidic conditions, nucleophilic attack of the thiol at the more-substituted α-carbon was favored while under basic conditions attack at the less-substituted β-carbon was more favored (Scheme 2).

**Scheme 3:**
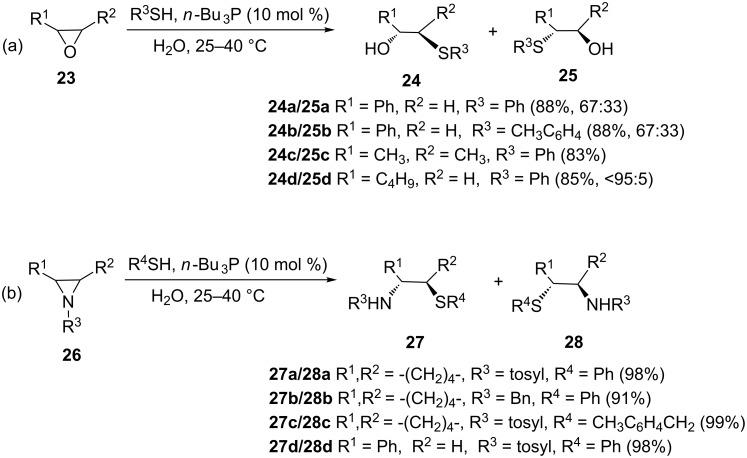
*n*-Bu_3_P-catalyzed thiolysis of epoxides and aziridines to provide the corresponding β-hydroxy and β-amino sulfides.

Prior to the report by Pizzo and co-workers, Fan and Hou had already indicated that *n*-Bu_3_P could catalyze the thiolysis of epoxides to provide the corresponding β-hydroxy sulfides [22]. Contrary to the reports of Pizzo and co-workers, in their hands the reaction proceeded faster at room temperature in the presence of water as a solvent (Scheme 3). For instance, under the Pizzo’s conditions the reaction provided 94% yield of β-hydroxy sulfide after two weeks of stirring while in the case of Fan and Hou the same product was obtained in 72% yield after only 12 h which suggested the importance of water in the reaction. As shown in Scheme 3, various epoxides and aziridines underwent reaction with various thiols to give the corresponding ring-opened β-hydroxy products in moderate to good yield.

The structure of the organophosphine was found to have a profound effect on the catalytic activity; some reactions were sluggish when tricyclohexylphosphine or triphenylphosphine were used as catalysts. The tributylphosphine acts as a nucleophilic trigger to attack, and thus initiates the reaction.

**Scheme 4:**
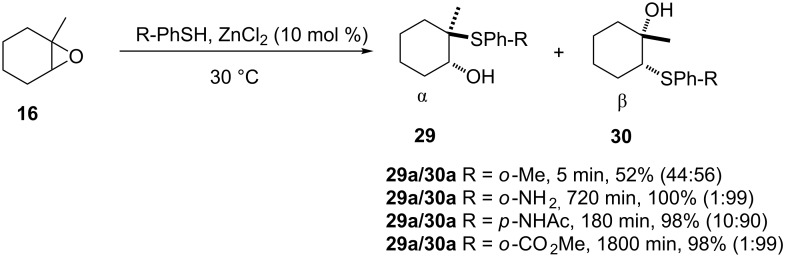
Zinc(II) chloride-mediated thiolysis of epoxides.

**3.1.1.3 Use of zinc salts.** In continuation of their effort to develop a general methodology for the thiolysis of epoxides, Pizzo et al. reported zinc(II) chloride-catalyzed thiolysis of epoxides in water at 30 °C as shown in Scheme 4 [23]. The reaction was compatible with a variety of epoxide substrates such as α- and β-substituted 1,2-epoxides, 2,3-epoxyalcohols as well as their OTMS, OTs and OPh derivatives. Reactions of 1-oxa-spiro[2.5]octane and 2-phenyloxirane (styrene oxide) with thiophenol both gave near quantitative yields of the hydroxy sulfides, with substitution occurring nearly exclusively at the sterically less hindered β-carbon of the epoxide.

The nature of the substituent on the aromatic ring of the thiophenol did not have any effect on the reaction except for those moieties that are strongly coordinating such as the amino and carboxyl groups. ZnCl_2_ was chosen as the preferred Zn(II) salt due to its low cost, the low catalyst loading required as well as recoverability and reusability without any significant loss of activity in terms of reaction time, selectivity and yield.

**Scheme 5:**
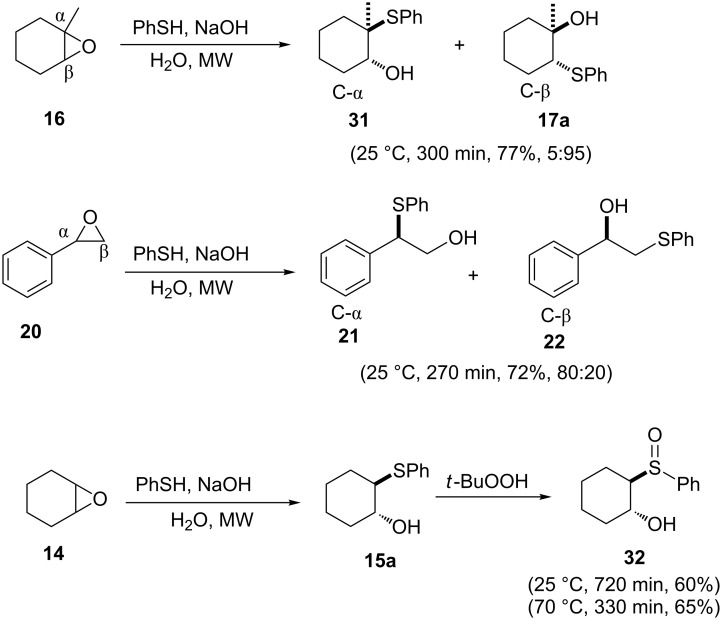
Thiolysis of epoxides and one-pot oxidation to β-hydroxy sulfoxides under microwave irradiation.

For reactions that are sluggish and mostly require heating, microwave irradiation is reported to significantly improve reaction rates, selectivities and yields much better than conventional heating [24]. Capitalizing on this, Pironti and Colonna achieved the thiolysis of epoxides in the presence of catalytic NaOH as shown in Scheme 5 [25]. The reaction was compatible with cycloalkane oxides, symmetrical epoxides and alkyl epoxides but only a single thiol was employed. In a subsequent one pot procedure, addition of *tert*-butyl hydroperoxide to the reaction mixture afforded β-hydroxy sulfoxide **32** in good yields.

**Scheme 6:**
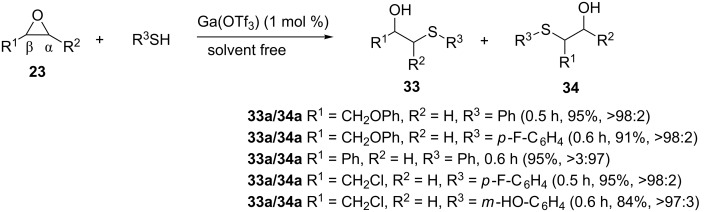
Gallium triflate-catalyzed ring opening of epoxides and one-pot oxidation.

In a similar fashion, Su et al. reported first the Ga(OTf)_3_ catalyzed regio- and stereoselective ring opening of epoxides **23** with thiols under solvent-free conditions as shown in Scheme 6 [26]. The reaction is compatible with both alkyl and arylthiols, with the nature of the substituent on the aromatic ring (electron donating/withdrawing) not having any influence on the reaction. Moreover, the reaction is compatible with epichlorohydrin (R^1^ = Cl) as well as alkyl (R^1^ = CH_3_) and aryloxy (R^1^ = ArO) substituents on the epoxide. Heterocyclic thiols gave products in moderate yields only, presumably due to the poor solubility of heterocyclic thiols in epoxides. However, the use of CH_3_NO_2_ as a solvent resulted in improved yields.

**Scheme 7:**
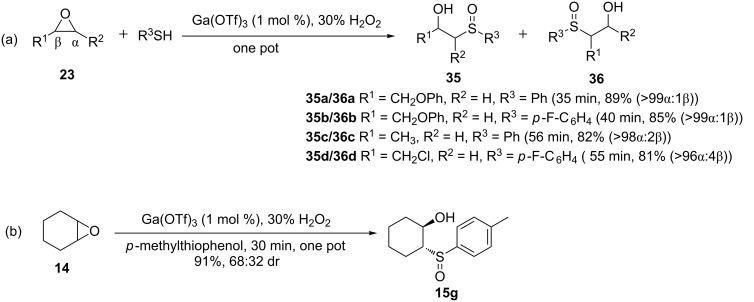
Thiolysis of epoxides and one-pot oxidation to β-hydroxy sulfoxides using Ga(OTf)_3_ as a catalyst.

Carrying out the one-pot thiolysis then oxidation with hydrogen peroxide proceeded uneventfully to give the corresponding β-hydroxy sulfoxides **35** and **36** in good to excellent yields (Scheme 7). Moreover, the reaction was found to be compatible with the less nucleophilic thiols such as *p*-fluorothiophenol and electron rich ones such as *p*-methylthiophenol, giving the corresponding products in very high yields. Unlike in the microwave irradiation protocol, the reaction of aliphatic thiols with epoxides proceeded smoothly to give excellent yields.

**Scheme 8:**
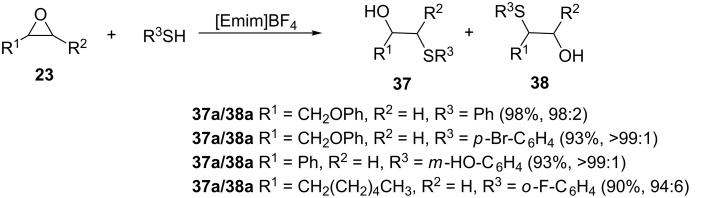
Ring opening of epoxide using ionic liquids under solvent-free conditions.

In another contribution, Su et al. carried out thiolysis of epoxides using ionic liquids under solvent free conditions [27]. The reaction is compatible with the presence of alkyl halides (R^1^ = CH_2_Cl), alkyls (R^1^ = CH_3_), aryloxy (R^1^ = ArO) and aryls on the epoxide ring as well as a wide range of thiophenols bearing a variety of substituents (Scheme 8). The nature of the substituent on the aromatic ring (electron withdrawing/donating) is reported to not have any influence on the outcome of the reaction. Unlike the ZnCl_2_-catalyzed protocol discussed previously, this method gave high chemo-selectivity with thiols bearing the nucleophilic groups such as NH_2_ and OH. In addition to its environmental benignity and ease of handling, the ionic liquid was reported to be reusable without any loss of activity after five runs.

**Scheme 9:**
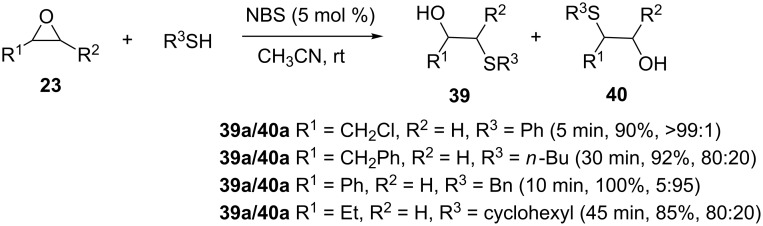
*N*-Bromosuccinimide-catalyzed ring opening of epoxides.

Another efficient catalyst for the synthesis of β-hydroxy sulfides is *N*-bromosuccinimide (NBS), as reported by Rostami and Jafari [28]. Various epoxides underwent reaction with different thiols (aromatic, benzylic, heterocyclic, cyclic and aliphatic (primary, secondary and tertiary)) in CH_3_CN at room temperature, to afford the corresponding β-hydroxy sulfides in good to excellent yields (Scheme 9). The reaction is compatible with alkyl halides (-CH_2_X), alkoxy (-CH_2_O), aryloxy (PhO), aryl (Ph) and alkyl (-CH_2_) substituents on the epoxide.

The virtues of this protocol are the low cost and ready availability of NBS, its moisture and air stability as well as low toxicity. The reaction is characterized by short reaction time, good to high product yield and high regioselectivity.

**Scheme 10:**
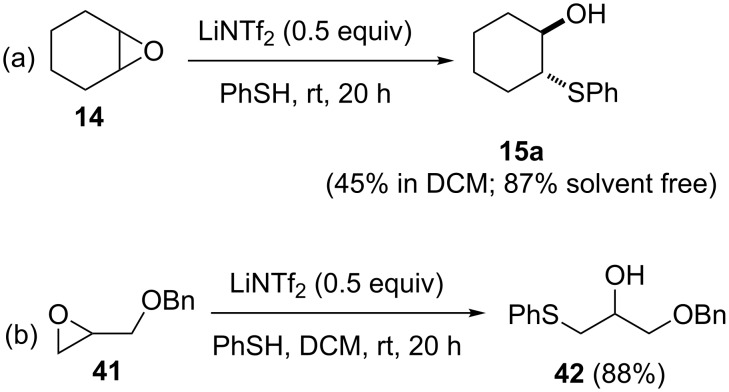
LiNTf_2_-mediated epoxide opening by thiophenol.

As an alternative to Lewis acids, Cossy and co-workers reported the use of LiNTf_2_ promoter for the thiolysis of epoxides [29]. The reaction provided better yields under solvent-free conditions than using dichloromethane as a solvent. Although a wide variety of epoxides, to varying levels of substitution and functionalization were tested using amines as nucleophiles, thiolysis was demonstrated with only two examples: using thiophenol as a nucleophile to open cyclohexene oxide (**14**) and benzyloxymethyloxirane (**41**, Scheme 10). Other than the mild reaction conditions, the added advantage of the protocol is that when LiNTf_2_ is used instead of Lewis acids, the work-up is easier as no vexatious emulsion was formed. However, the need for 0.5 equiv of the promoter and the sluggishness (20 h) of the reaction make the method less favorable when compared to other Lewis acid catalyzed protocols.

**Scheme 11:**
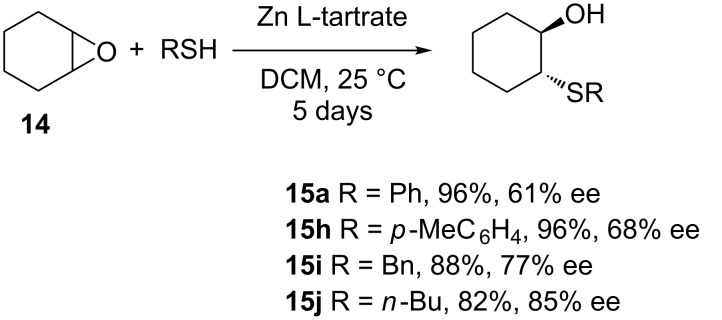
Asymmetric ring-opening of cyclohexene oxide with various thiols catalyzed by zinc L-tartrate.

Many asymmetric versions of epoxide opening by thiols assisted by chiral catalysts have been reported and are part of several reviews [30–36]. In 1985, Yamashita and Mukaiyama demonstrated the efficacy of zinc L-tartrate as a heterogeneous chiral Lewis acid catalyst for the asymmetric ring opening of cyclohexene oxide with alkyl- and arylthiols [37]. The optimal conditions were found to be 10 mol % of the catalyst relative to cyclohexene oxide, at room temperature, in dichloromethane as the solvent (Scheme 11). More polar solvents such as DMF retarded the reaction due to their tight coordination to the catalyst whilst non-polar solvents such as cyclohexane resulted in poor enantioselectivity probably due to coordination of the product β-hydroxy sulfide. The protocol was, however, limited to unsubstituted cyclohexene oxide, and as its greatest weakness it required long reaction times (5 days).

**Scheme 12:**
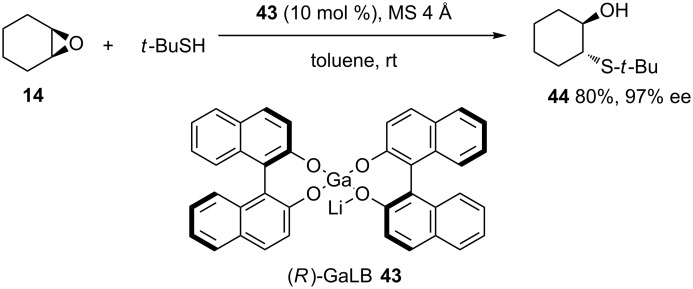
Catalytic asymmetric ring opening of symmetrical epoxides with *t*-BuSH catalyzed by (*R*)-GaLB (**43**) with MS 4 Å.

In 1997, Shibasaki and colleagues reported a gallium-lithium bis(binaphthoxide) complex **43**, easily prepared from GaCl_3_, (*R*)-binaphthol and butyllithium in THF, as a catalyst for the asymmetric opening of symmetrical epoxides in the presence of 4 Å molecular sieves [38]. Whilst yields and enantioselectivities were impressive, only *t*-BuSH was used as the nucleophile and reaction times were quite variable, ranging from 9 to 137 hours (Scheme 12).

**Scheme 13:**
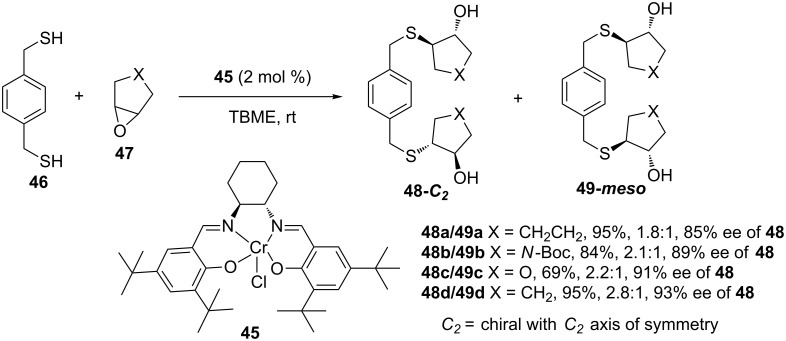
Asymmetric ring opening of *meso*-epoxides by *p*-xylenedithiol catalyzed by a (*S,S*)-(salen)Cr complex.

A year later (1998), Wu and Jacobsen reported the use of (salen)Cr(III) complex **45** as a catalyst for the asymmetric ring opening of *meso*-epoxides with *p*-xylenedithiol [39]. The reactions were performed at room temperature in *tert*-butyl methyl ether (TBME) as the solvent, with rigorous exclusion of oxygen, giving the desired products in excellent yields and enantioselectivities, over one to four days of reaction time (Scheme 13).

In the same year, Hou and co-workers reported the use of (salen)Ti(IV) in the ring opening of *meso*-epoxides with various thiophenols and benzylmercaptan, in yields of up to 93% over 2–24 hours, albeit with lower enantioselectivities [40]. The use of salen complexes inspired others, with Zhou et al. in 2006 reporting the same (salen)Ti(IV) complexes as Wue et al. and variations thereof, prepared in situ, to be useful in thiolysis of epoxides with dithiophosphorus acid as the nucleophile, in toluene at room temperature, under an inert atmosphere for 20 minutes. Product yields ranged from 67–92%, with enantioselectivities of 21–73% [41]. In 2009, Sun et al. reported heterobimetallic gallium–titanium variations of the same salen complexes as excellent catalysts for the thiolysis and selenolysis of *meso*-epoxides, giving the desired β-hydroxy sulfides and selenides in near quantitative yields and generally very high enantioselectivities [42–43].

**Scheme 14:**
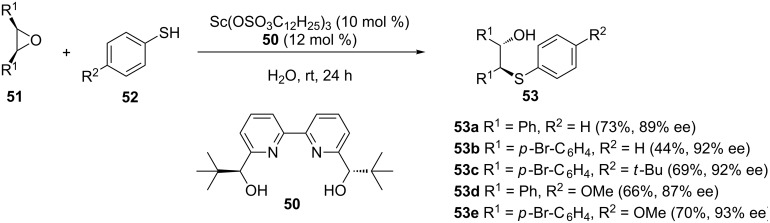
Desymmetrization of *meso*-epoxide with thiophenol derivatives.

Using the chiral bipyridine ligand **50** and Sc(OSO_3_C_12_H_25_)_3_ as a catalyst, the asymmetric thiolysis of *meso-*epoxides **51** in neat water was achieved to provide the corresponding β-hydroxy sulfides in moderate to good yields with high enantioselectivities (Scheme 14) [44]. The reaction was also found to be compatible with aromatic N-heterocycles and alcohol nucleophiles to afford the corresponding products in moderate to good yields with high to excellent enantioselectivities [44].

The use of water as a solvent by Kobayashi and co-workers is notable; this was the first enantioselective thiolysis of a *meso*-epoxide in pure water as a solvent. Since this report, several epoxide thiolysis reactions in water have been reported and are part of several reviews [45–46].

**Scheme 15:**
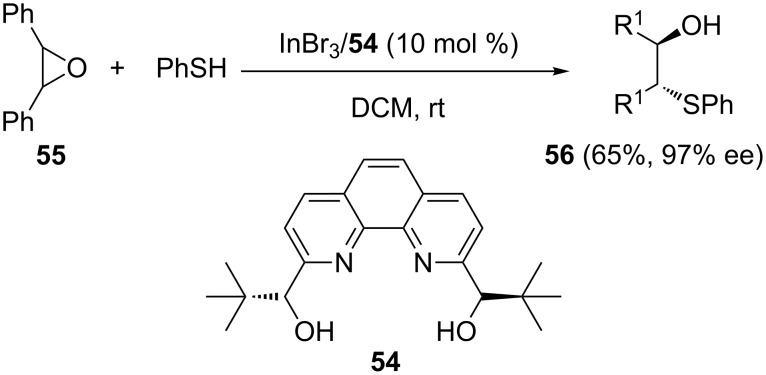
Enantioselective ring-opening reaction of *meso*-epoxides with ArSH catalyzed by a *C*_2_-symmetric chiral bipyridyldiol–titanium complex.

Schneider and co-workers reported the same scandium–bipyridine complex as excellent catalyst for the thiolysis and selenolysis of *meso*-epoxides to give the corresponding β-hydroxy derivatives in good yields and high enantioselectivities, in dichloromethane as the solvent [47–48]. As an extension to this work, a novel chiral 2,9-disubstituted-1,10-phenanthroline **54** was prepared by the same group and its utility in the enantioselective ring-opening of *cis*-stilbene oxide with aniline and thiophenol was demonstrated. The reaction provided the corresponding β-hydroxy derivatives in good yields and excellent enantioselectivities (Scheme 15) [49].

**Scheme 16:**
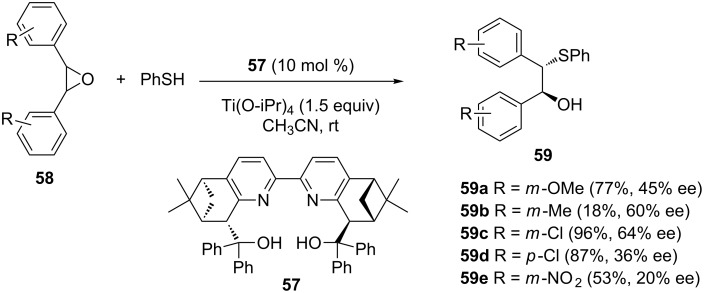
Enantioselective ring-opening reaction of stilbene oxides with ArSH catalyzed by a *C*_2_-symmetric chiral bipyridyldiol–titanium complex.

Bipyridine as a scaffold in ligand design was also adopted by Chen and Chen, who reported the bipyridine-pinene ligand **57** in combination with Ti(O-iPr)_4_ as an effective catalyst in the asymmetric ring opening of *meso*-stilbene oxides with thiophenol, in acetonitrile as the solvent, for 30 hours, at room temperature under a nitrogen atmosphere [50]. Product yields ranged from 18 to 96%, but in moderate enantioselectivities of 20–64% ee (Scheme 16).

**Scheme 17:**
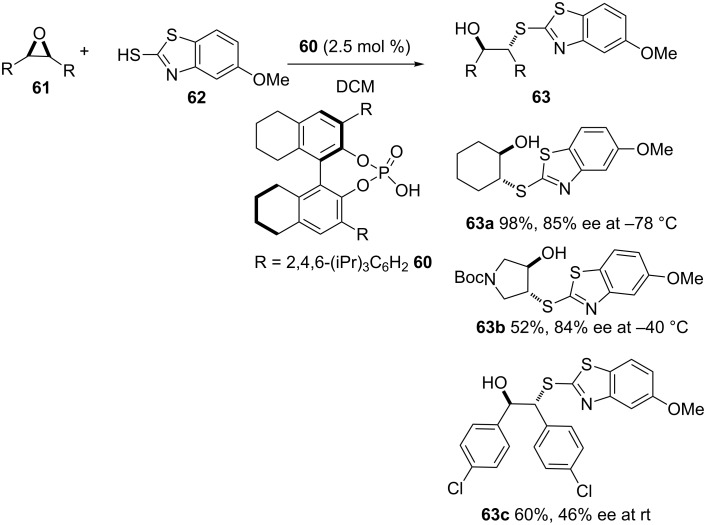
Asymmetric desymmetrization of *meso-*epoxides using BINOL-based Brønsted acid catalysts.

Sun and co-workers reported the use of BINOL-based Brønsted acid catalysts such as TRIP **60** for the asymmetric thiolysis of *meso*-epoxides with benzothiazoles **62** as nucleophiles as shown in Scheme 17 [51]. The reaction provides moderate to excellent yields and 46–85% ee. Although the reaction was reported to be compatible with various cyclic and acyclic epoxides, it is limited to the use of benzothiazoles as nucleophiles. In most cases, the reaction required very low temperatures that ranged from –78 °C to room temperature. The use of amines, alcohols as well as alkyl and arylthiols as nucleophiles failed to provide the corresponding products.

**Scheme 18:**
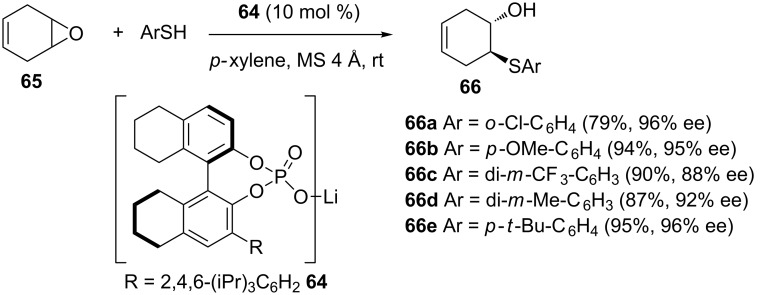
Lithium-BINOL-phosphate-catalyzed desymmetrization of *meso*-epoxides with aromatic thiols.

A year later, Antilla and co-workers found lithium-binol phosphate **64** to be an efficient catalyst for the desymmetrization of *meso*-epoxides with aromatic thiols as shown in Scheme 18 [52]. The reactions were carried out in *p*-xylene as the solvent, at room temperature, for 48 hours, in the presence of 4 Å molecular sieves and 10–20 mol % of the catalyst, yielding the desired β-hydroxy sulfides in yields of 67–98%, and enantioselectivities of 75–96% (Scheme 18). The reaction showed a broad substrate scope in terms of epoxides and aromatic thiols.

**Scheme 19:**
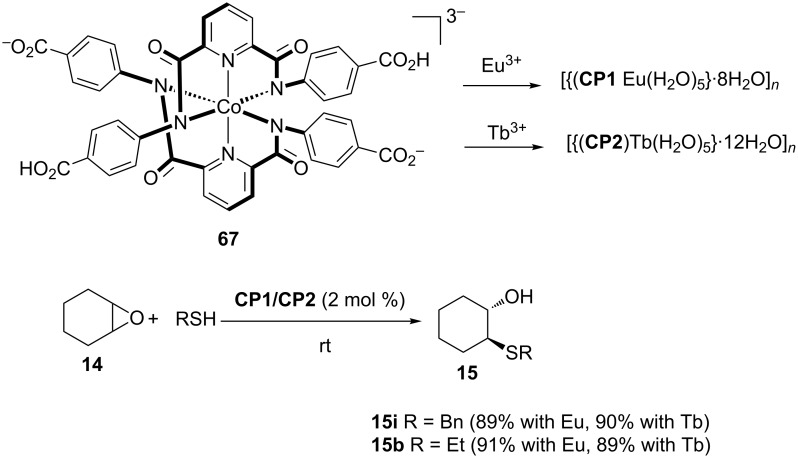
Ring-opening reactions of cyclohexene oxide with thiols by using **CPs 1**-Eu and **2**-Tb.

Most recently, Kumar et al. synthesized Eu^3+^- and Tb^3+^-based coordination polymers (CPs) which function as reusable heterogeneous catalysts for ring-opening reactions utilizing amines, alcohols, thiols, and azides as the nucleophiles [53]. The Eu^3+^ and Tb^3+^ versions were prepared by metal exchange in methanol, between the cobalt complex **67** and the triflate salts of the rare earths, giving the desired complexes **CP1** and **CP2** in 83% and 86% yield, respectively. The solvent free thiolysis of cyclohexene oxide stereoselectively provided the *trans*-β-hydroxy sulfide as the only product whereas styrene oxide afforded the exclusive regioselective terminal alcohol products (Scheme 19). Both complexes could be easily recovered by filtration and were reused up to five times without any significant drop in activity. Mechanistic studies substantiated Lewis-acid-catalyzed activation of the epoxide during the reaction.

**Scheme 20:**
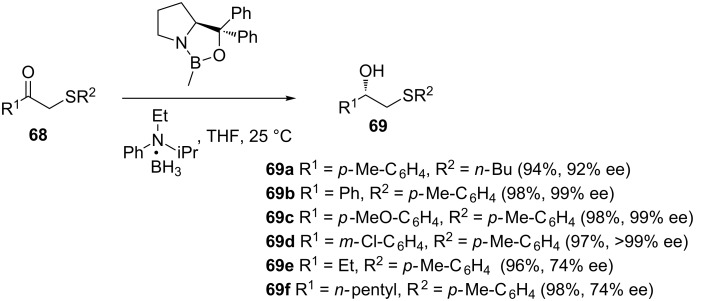
CBS-oxazaborolidine-catalyzed borane reduction of β-keto sulfides.

In addition to the asymmetric ring opening of *meso-*epoxides, other alternative chemical and biocatalytic methodologies have also been explored. Cho and co-workers reported a CBS-oxazaborolidine-catalyzed asymmetric borane reduction of β-keto sulfides **68** for the synthesis of β-hydroxy sulfides with high enantiomeric purity (Scheme 20) [54–55]. The enantioselectivity was highly dependent on the nature and bulkiness of the substituents on both ends of the carbonyl group. While the reduction of aromatic β-keto sulfides provided excellent enantioselectivity, the reduction of the corresponding alkyl β-keto sulfides gave low enantioselectivity.

**Scheme 21:**
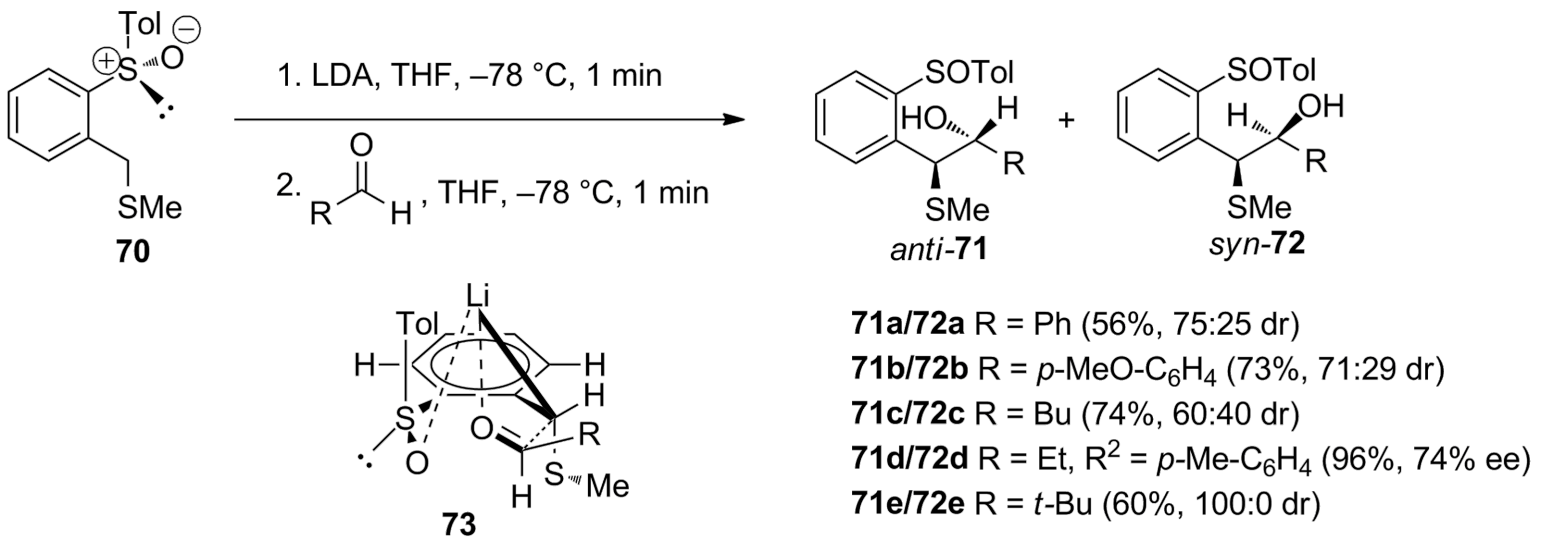
Preparation of β-hydroxy sulfides via connectivity.

Ruano and co-workers reported the asymmetric synthesis of β-hydroxy sulfides via connectivity of a suitably substituted benzylic carbanion with various aldehydes as shown in Scheme 21 [56]. It involved the reaction of a pre-prepared enantiopure toluene **70** with aromatic as well as aliphatic aldehydes in the presence of LDA. The stereoselectivity was in favor of the *anti*-diastereoisomer and was found to be dependent on the size of the aldehyde. It was proposed to proceed via the formation of the transition state **73** to avoid steric interaction between the R group of the aldehyde and the SMe of the toluene precursor, hence, led to *anti* selectivity.

**Scheme 22:**
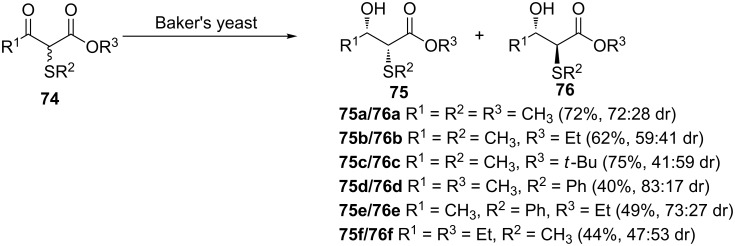
Baker’s yeast-catalyzed reduction of sulfenylated β-ketoesters.

The biocatalytic reduction of α-sulfenyl-β-ketoesters **74** using Baker’s yeast was reported by Fujisawa and co-workers in 1984 as depicted in Scheme 22 [57]. Coincidentally, the presence of the sulfenyl group at the α-position of the carbonyl center was found to be crucial for the stereoselectivity. Despite the sharp advancement in the field of biotechnology in the last decade, the application of biocatalysis for the asymmetric synthesis of β-hydroxy sulfides still remains unexplored.

**Scheme 23:**
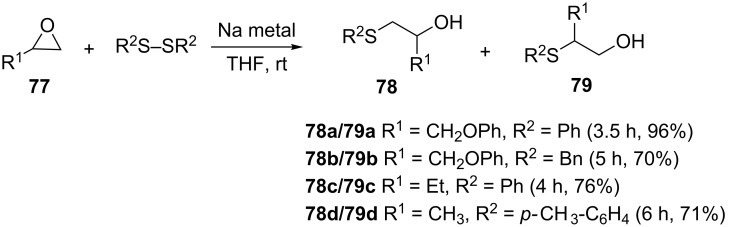
Sodium-mediated ring opening of epoxides.

**3.1.2 Disulfides as nucleophiles.** Whilst thiols are excellent nucleophiles and can open epoxides under a variety of conditions as detailed above, they can be awfully odoriferous and unpleasant to work with [58]. As an alternative, Soleiman-Beigi et al. reported an epoxide opening using disulfides in the presence of sodium metal in THF at room temperature (Scheme 23) [59]. The reaction is compatible with different kinds of epoxides possessing substituents such as alkyl, aryloxy and phenylepoxy, and the nature of the substituent (electron donating/withdrawing) on the aromatic ring of the disulfide was reported to have no effect on the reaction (Scheme 23). Despite using a very reactive metal, the advantage of this method is that there is no use of any extra acidic catalyst for activating the epoxide ring, and moderate to good yields of products and remarkable regioselectivity in most cases are obtained.

**Scheme 24:**
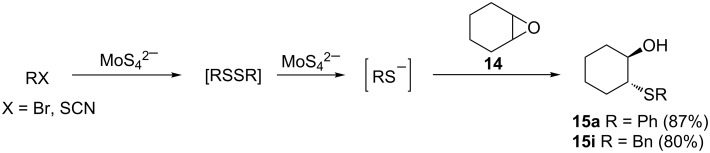
Disulfide bond cleavage-epoxide opening assisted by tetrathiomolybdate.

Devan et al. reported a one-pot, multistep tetrathiomolybdate-assisted epoxide ring opening with masked thiolates and selenoates [60]. Treatment of epoxides with in situ-generated disulfides in the presence of benzyltriethylammonium tetrathiomolybdate, [C_6_H_5_CH_2_NEt_3_]_2_MoS_4_ over 4–12 hours produced the desired hydroxy sulfides in yields of 48–90% (Scheme 24).

**Scheme 25:**
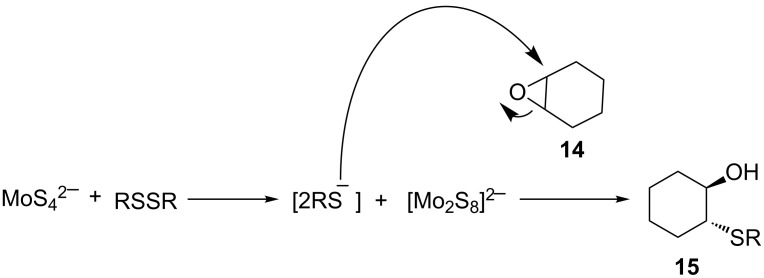
Proposed reaction mechanism of disulfide bond cleavage-epoxide opening assisted by tetrathiomolybdate.

The authors proposed that the reaction proceeds via a reductive cleavage of the in situ-generated disulfide (RSSR). The resulting thiolate then undergoes thiolysis of the epoxide to provide the corresponding β-hydroxy sulfide as shown in Scheme 25.

In addition to disulfides, other odorless thiol equivalents have also been employed as nucleophiles for the thiolysis of epoxides and provided comparable results. Examples include ketene-*S,S-*acetals (2-[bis(alkylthio)methylene]-3-oxo-*N*-*o*-tolylbutanamides) [61] and *S*-alkylisothiouronium salts [62].

#### 3.2 Synthesis of β-hydroxy sulfides via difunctionalization of alkenes

Although the synthesis of β-hydroxy sulfides via the thiolysis of epoxides is important, its Achilles’ heel is the need for the preparation of an epoxide precursor and undesirable side products via rearrangement of epoxides. Attempts to mitigate against these limitations include the direct 1,2-hydroxysulfenylation of alkenes with thiols or disulfides using various catalyst systems, as detailed below.

**Scheme 26:**
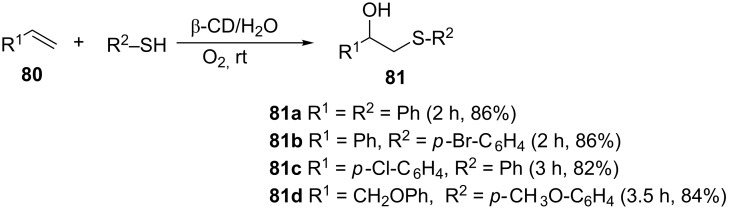
Cyclodextrin-catalyzed difunctionalization of alkenes.

**3.2.1 Cyclodextrin as a catalyst.** Rao and co-workers reported the use of β-cyclodextrin as a catalyst for the hydroxysulfurization of alkenes with thiophenols in neat water under atmospheric oxygen as shown in Scheme 26 [63]. As opposed to the usual acid-base type of catalysis, β-cyclodextrin having hydrophobic cavities catalyze reactions via formation of host–guest complexes by non-covalent bonding [64]. This mode of catalysis is similar to the way enzymes mediate biochemical reactions [64]. The use of water as a solvent and the non-toxicity of the cyclodextrin catalyst make the method attractive. This method, like the previously reported protocols, is also compatible with both electron-donating and electron-withdrawing possessing styrenes and thiols (Scheme 26). The superiority of the protocol when compared with the other methodologies is its compatibility with olefins such as the less substituted cyclohexene and phenoxy alkenes.

**Scheme 27:**
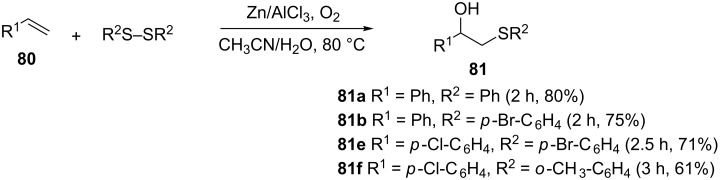
Zinc-catalyzed synthesis of β-hydroxy sulfides from disulfides and alkenes.

**3.2.2 The use of zinc/aluminium chloride.** Movassagh et al. reported another simple, general and highly regioselective one pot synthesis of β-hydroxy sulfides in good yields from the reaction of styrenes and disulfides using Zn/AlCl_3_ as a catalyst in aqueous CH_3_CN at 80 °C, in the presence of oxygen as oxidant (Scheme 27) [65]. Similar to the cyclodextrin-catalyzed methodology, the protocol is compatible with the presence of both electron-donating and electron-withdrawing substituents on the styrene and the disulfide but the reaction was found to be slightly slower. The presence of aerial oxygen is crucial to effect the reaction since in its absence, the addition of hydrogen and –SR across the double bond ensues, yielding the undesired ArCH_2_CH_2_SAr according to Markovnikov's rule.

**Scheme 28:**
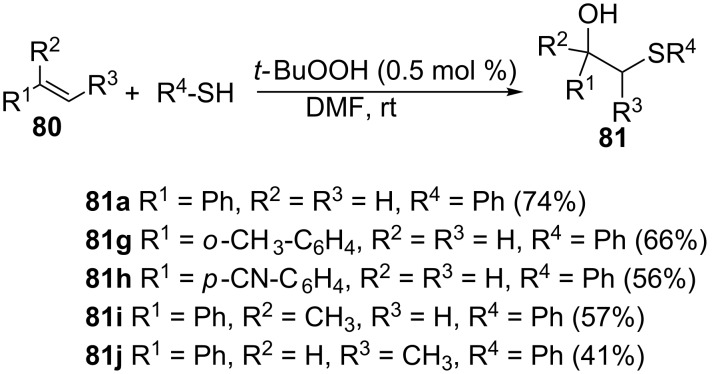
*tert*-Butyl hydroperoxide-catalyzed hydroxysulfurization of alkenes.

**3.2.3 The use of *tert*-butyl hydroperoxide.** Zhou et al. reported the hydrosulfurization of alkenes with thiols for the direct synthesis of β-hydroxy sulfides, with a catalytic amount of *tert*-butyl hydroperoxide (TBHP) as an initiator (Scheme 28) [66]. Even styrenes bearing powerful electron withdrawing groups such as nitriles (CN), trifluoromethyl (CF_3_) and ester groups (COOCH_3_) gave the corresponding β-hydroxy sulfides, albeit in lower yields (Scheme 28). The reaction is also compatible with α- or β*-*substituted styrenes, unlike the other protocols, with *α*-methylstyrene (**81i**) and β-methylstyrene (**81j**) giving the corresponding β-hydroxy sulfides in 57 and 41% yields, respectively. α-Bromostyrene was also tested, but unfortunately gave an oxosulfide as the only product, in a low (44%) yield. In general, product yields were lower than those from the reaction of the corresponding unsubstituted styrenes. This was ascribed to steric encumbrance exerted by substituents located at the α- or β-position on the incoming thiol nucleophile. The arylthiols bearing chloro and fluoro substituents gave the expected β-hydroxy sulfides in moderate yields while the much deactivated nitro-thiophenol did not lead to any product formation. The protocol allowed easy scale-up to gram quantities.

**Scheme 29:**
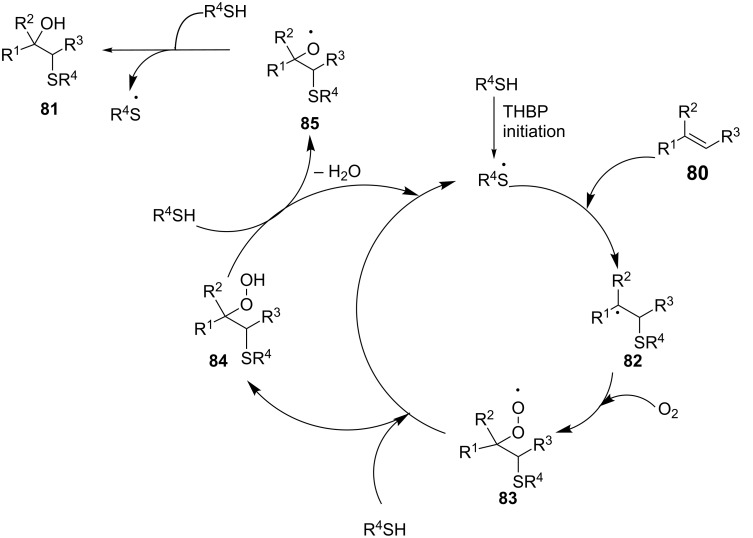
Proposed mechanism of the radical hydroxysulfurization.

The results strongly supported a free radical mechanism adumbrated in Scheme 29 [66]. The thiyl radical resulting from the initial reaction of TBHP and thiophenol selectively adds to the terminal end of the C=C bond of **80** to form intermediate radical **82**. Oxidation with O_2_ leads to the formation of peroxy radical **83**, which abstracts a hydrogen radical from the thiophenol to form β-peroxysulfide **84**. Cleavage of the O–O bond of the peroxide followed by protonation liberates the desired β-hydroxy sulfide **81**. This methodology is quite general considering the number of substituents used in both the styrenes and thiophenols.

**Scheme 30:**
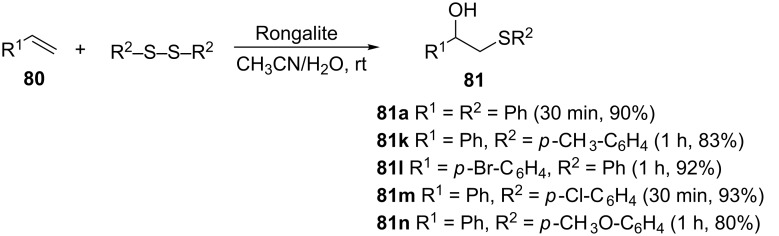
Rongalite-mediated synthesis of β-hydroxy sulfides from styrenes and disulfides.

**3.2.4 Rongalite catalysis.** Yadav et al. reported a very efficient one pot protocol of β-hydroxy sulfides synthesis from disulfides and styrenes in open air, at room temperature, using inexpensive Rongalite [sodium formaldehyde sulfoxylate (Na(HOCH_2_SO_2_)·2H_2_O)], as a promoter (Scheme 30) [67].

The protocol is compatible with styrenes and disulfides possessing electron-donating as well as electron-withdrawing substituents to provide the corresponding β-hydroxy sulfides in quantitative yields, with styrenes or disulfides bearing electron-withdrawing groups giving slightly better yields in shorter reaction times than those with electron-donating groups. Whilst this method offers a one-pot, transition metal-free and odorless approach to β-hydroxy sulfides utilizing atmospheric oxygen, an environmentally benign and cheap oxidant, its chief drawback is the incompatibility with aliphatic alkenes and dialkyl disulfides to provide the corresponding β-hydroxy sulfides.

**Scheme 31:**
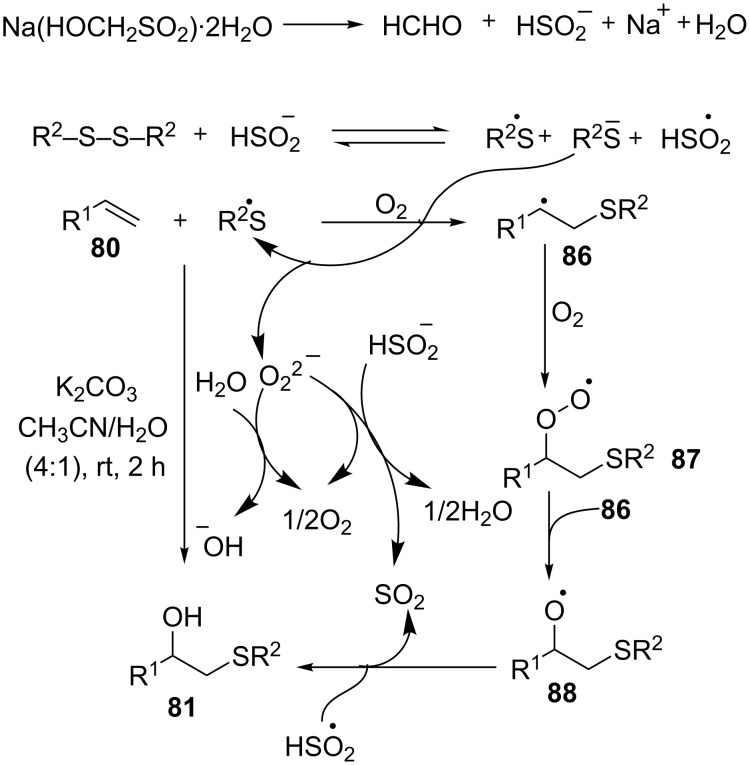
Proposed mechanism of Rongalite-mediated synthesis of β-hydroxy sulfides from styrenes and disulfides.

The authors proposed the mechanism depicted in Scheme 31 [67]. The hydroxysulfurization commences with the formation of a thiyl radical that is generated by the heterolytic cleavage of the disulfide upon reaction with HSO_2_^−^. The thiyl radical then adds to the double of the alkene to form radical **87**. This intermediate undergoes oxidation followed by decomposition to provide the alkoxy radical intermediate **88**, which then affords the β-hydroxy sulfide **81** upon protonation.

The considerably stronger S–S bonds of the dialkyl disulfides and the lower stability of the expected alkyl radicals than the corresponding diaryl disulfides and benzyl radicals, respectively, are presumed to be the reasons behind the failure of the reaction to work with aliphatic alkenes [67].

#### 3.3. Synthesis of masked β-hydroxy sulfides via difunctionalization of alkenes

Alkenes are versatile substrates offering bountiful opportunities to a multitude of derivatives, including β-hydroxy sulfides. One popular route to the latter is the 1,2-acetoxysulfenylation of alkenes using various catalysts has proven an attractive indirect route to β-hydroxy sulfides, masked as their acetates, as detailed below.

**Scheme 32:**
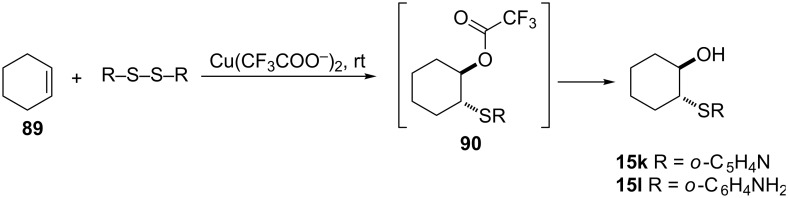
Copper(II)-catalyzed synthesis of β-hydroxy sulfides **15e**,**f** from alkenes and basic disulfides.

**3.3.1 Acetoxysulfenylation using copper acetate.** Bewick et al. reported a copper(II)-catalyzed synthesis of β-hydroxy sulfides from the reaction of cyclohexene/open-chain olefin with the basic disulfides, namely: 2,2’-dipyridyl disulfide and bis(2-aminophenyl) disulfide [68]. The intermediate **90** hydrolyzes in the work-up to provide the β-hydroxy sulfide **15** as shown in Scheme 32 [68]. The protocol is a supplement to the lead(IV)-promoted addition of disulfides to alkenes in dichloromethane–trifluoroacetic acid to give, after hydrolysis, products of hydroxysulfination of simple diaryl and dialkyl sulfides. The reaction is proposed to proceed via formation of a copper complex by coordination of the copper to the sulfur and nitrogen atoms of the disulfide. The complexation is accompanied by an increase in the sulfur–sulfur bond length and assists the cleavage of this bond by the attacking nucleophile. Due to the absence of this kind of chelation effect, the copper(II) acetate fails to catalyze the addition of diphenyl disulfide to alkenes under the same reaction conditions.

**Scheme 33:**
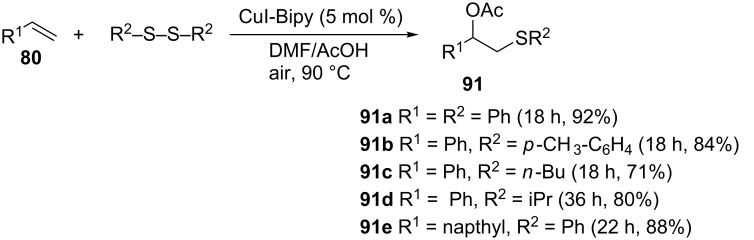
CuI-catalyzed acetoxysulfenylation of alkenes.

**3.3.2 Acetoxysulfenylation using copper iodide-bipyridine as a catalyst.** Taniguchi reported a copper(II) iodide-catalyzed 1,2-acetoxysulfenylation of alkenes using disulfides and acetic acid as substrates at 90 °C in open air as depicted in Scheme 33 [69]. This regioselective reaction gave the corresponding 1,2-acetoxysulfides in reasonable yields. Unlike a copper(II) acetate-catalyzed reaction which requires long reaction times, the copper(II) iodide catalyst enables the use of both diaryl and dialkyl disulfides to provide the corresponding masked β-hydroxy sulfides.

**Scheme 34:**
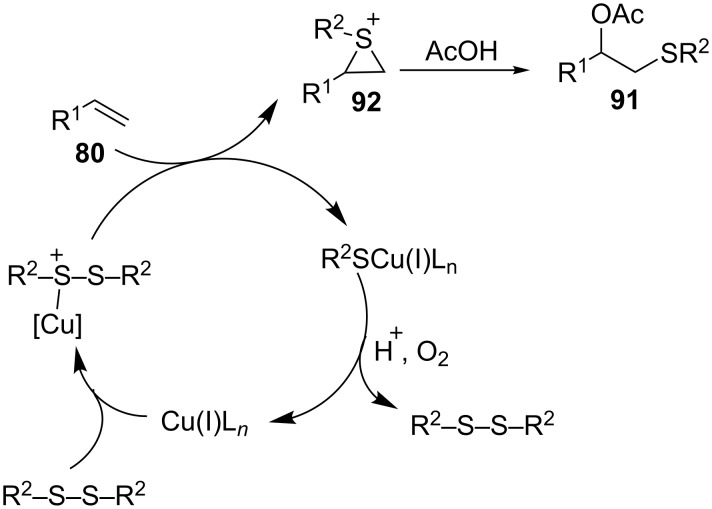
CuI-catalyzed acetoxysulfenylation reaction mechanism.

The proposed mechanism is depicted in Scheme 34 [69]. The reaction commences with the complexation/activation of the disulfide by the CuI catalyst. The nucleophilic alkene then attacks the electrophilic site of the disulfide to provide the sulfonium ion **92**, which then undergoes regioselective ring opening to afford the 1,2-acetoxysulfide **91**. The R^2^SCu(I)L*_n_* that was produced in the previous step is oxidized to regenerate the active catalyst and a disulfide that is re-introduced into the catalytic cycle. Several copper salts (CuCl, CuCl_2_, CuOAc and Cu(OAc)_2_) were evaluated as part of optimizing the reaction conditions and they all provided poor yields.

**Scheme 35:**
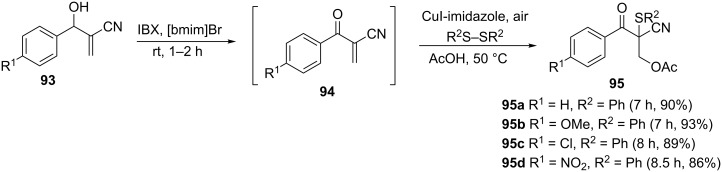
One-pot oxidative 1,2-acetoxysulfenylation of Baylis–Hillman products.

**3.3.3 Acetoxysulfenylation of Baylis–Hillman alcohols.** Yadav and Aswathi reported a regioselective CuI-imidazole-catalyzed 1,2-acetoxysulfenylation of Baylis–Hillman products at 50 °C under an oxygen atmosphere as shown in Scheme 35 [70]. The β-ketomethylene **94** substrate was generated in situ from the oxidation of Baylis–Hillman product **93** using a hypervalent iodine reagent (iodoxybenzoic acid, IBX) in an ionic liquid.

**Scheme 36:**
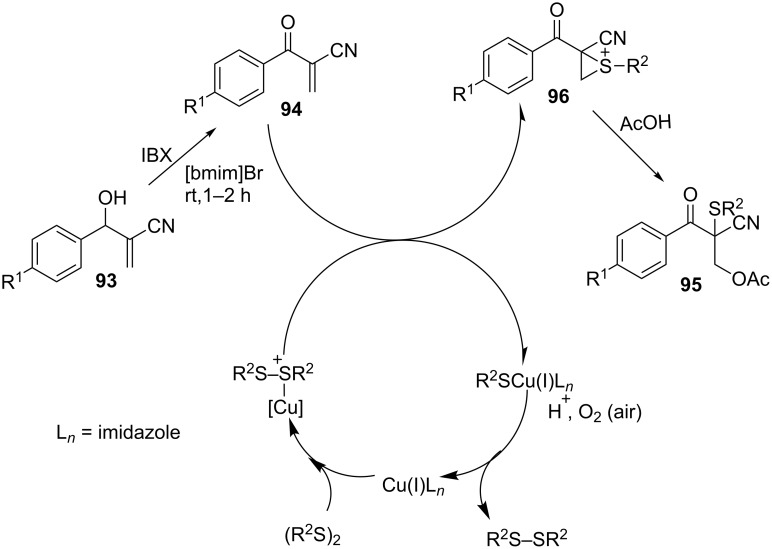
Proposed mechanism for the oxidative 1,2-acetoxysulfination of Baylis–Hillman products.

The reaction works quite well with both electron-donating and electron-withdrawing substituents on the Baylis–Hillman alcohols. The protocol is also compatible with both diaryl and dialkyl sufides. What is even more interesting about this methodology is the recyclability and reusability of the ionic liquid for up to four times without any loss of efficiency. The proposed mechanism of the reaction is depicted in Scheme 36 [70]. The reactive R^2^S^+^ that is generated from the reaction of the CuI catalyst and the disulfide reacts with the β-ketomethylene **94** to form sulfonium intermediate **96**. Regioselective ring opening of the sulfonium ion then provides the β-hydroxy sulfide **95**. The R^2^SCu(I)L*_n_* generated in the reaction is oxidized to form the disulfide and regenerates the Cu(I)L*_n_* catalyst.

**Scheme 37:**
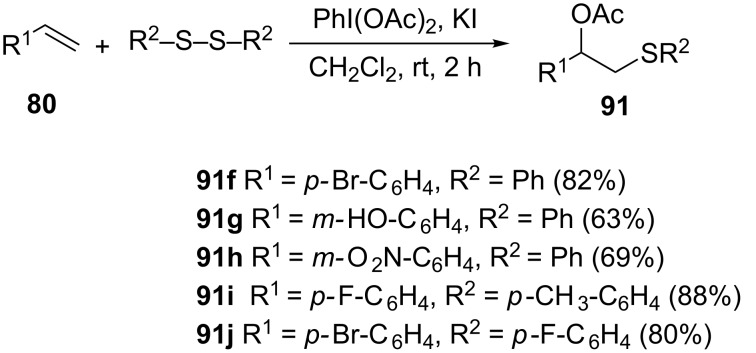
1,2-Acetoxysulfenylation of alkenes using DIB/KI.

**3.3.4 Acetoxysulfenylation of alkenes using DIB/KI.** Muangkaew et al. reported a convenient method for 1,2-acetoxysulfenylation of alkenes using diaryl disulfide promoted by diacetoxyiodobenzene (DIB) and KI as shown in Scheme 37 [71].

The method is compatible with styrenes possessing various substituents, namely: halides, methoxy, hydroxy, nitro, ester and alkyl halides. Similarly, the nature of the substituents on the aromatic ring of the disulfide partner was found to have an insignificant effect on the outcome of the reaction. Just like in the case of the styrene analog, both electron-donating and electron-withdrawing substituents were tolerated. Unlike the methods discussed previously, this method catalyzed the 1,2-acetoxysulfenylation of aliphatic alkenes in moderate to good yields but the products are obtained as a mixture of two regioisomers. In the case of cyclohexene, the reaction gave the corresponding 1,2-acetoxysulfide product as a single isomer. Similar results were reported by Marakalala and Kinfe where a catalytic amount of iodine was used instead of stoichiometric amount of KI [72].

**Scheme 38:**
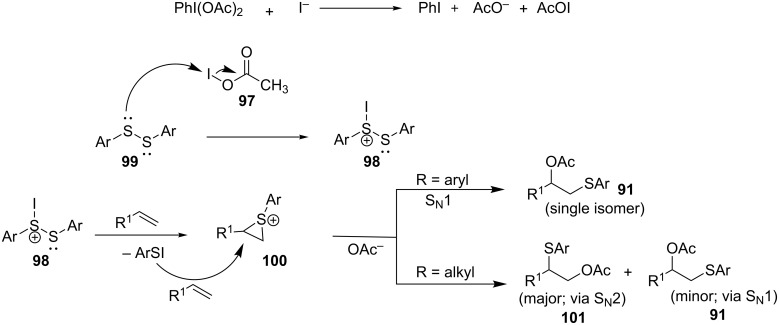
Proposed reaction mechanism of the diacetoxyiodobenzene (DIB) and KI-mediated 1,2-acetoxysulfenylation of alkenes.

The proposed mechanism is depicted in Scheme 38 [71]. The DIB reacts with the iodide yielding acetylhypoiodite (**97**, AcOI) as an intermediate which according to the authors readily reacts with the disulfide **99**. The thus formed electrophilic thionium intermediate **98** upon reaction with the alkene forms a cyclic sulfonium ion **100** and arylsulfenyl iodide (ArSI). This in situ generated ArSI can transfer a second equivalent of electrophilic sulfide to the alkene to form a cyclic sulfonium ion **100** which upon reaction with an acetate undergoes regioselective ring opening to provide the 1,2-acetoxysulfide product **91**. The results obtained (1,2-*trans-*isomers) when cyclohexene being used as an alkene substrate are indicative of the formation of the cyclic sulfonium intermediate **100**.

The PhI(OAc)_2_/KI reagent system is attractive as it does not require the use of transition metals. However, the need for stoichiometric amount of the KI promoter (2 equivalents of KI is required relative to a diaryl disulfide substrate) calls for the development of new alternative catalytic methodologies.

#### 3.4 Thiofunctionalization of unactivated alkenes

**Scheme 39:**
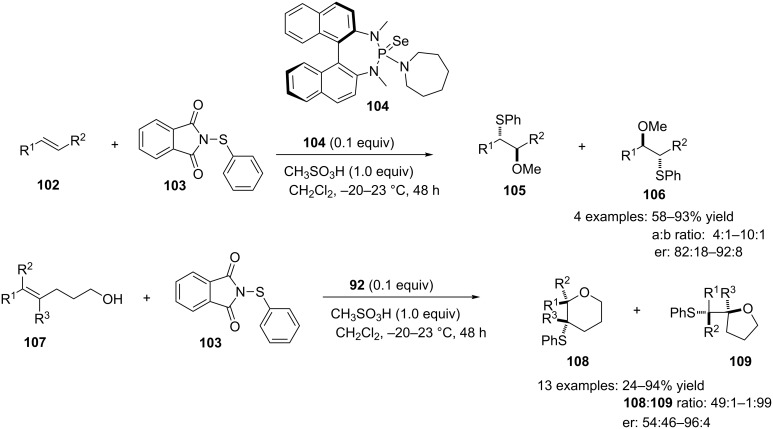
Catalytic asymmetric thiofunctionalization of unactivated alkenes.

A few years ago, Denmark and co-workers revealed an impressive catalytic asymmetric thiofunctionalization of unactivated alkenes **102** with the commercially available arylsulfenylating agent *N*-(phenylthio)phthalimide (**103**) using selenophosphoramide **104** as a catalyst in the presence of methanesulfonic acid [73] (Scheme 39). The reaction was found to be useful in both intra- and intermolecular thioetherification to yield the regioisomers in ratios that were dependent on the steric bulk of the substituents on the alkene (Scheme 39).

**Scheme 40:**
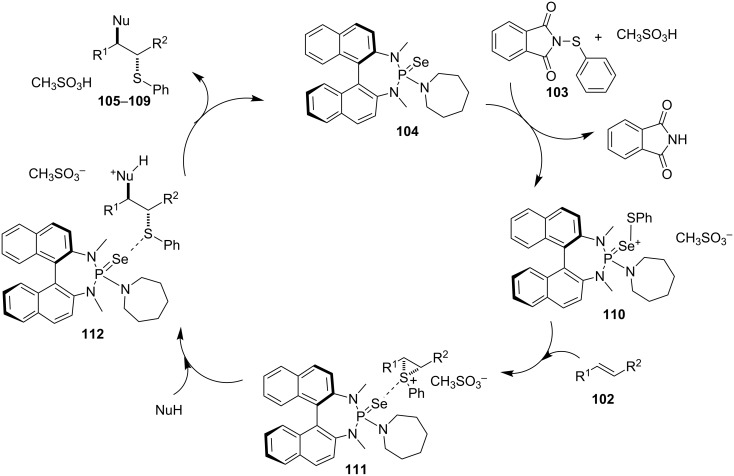
Proposed catalytic cycle for asymmetric sulfenofunctionalization.

The reaction was proposed to proceed via initial transfer of the thiolate group from succinimide **103** to the chiral pre-catalyst **104** to generate the active species **110** (Scheme 40). Reaction of the alkene with the active species **110** results to the formation of the thiiranium intermediate **111**, which then undergoes a stereoselective nucleophilic addition to provide the thioethers **105**–**109** (Scheme 40) [73].

#### 3.5 Other synthetic routes to β-hydroxy sulfides

**Scheme 41:**
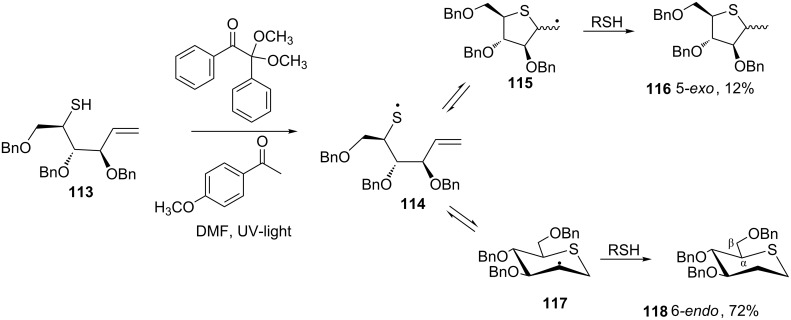
Synthesis of thiosugars using intramolecular thiol-ene reaction.

Incorporation of amine, silyl and sulfide functional groups into C–C multiple bonds offers an attractive route to appropriately functionalized substrates. Inter- and intramolecular thiol-ene reaction is a highly efficient, free-radical mediated “click” process with diverse applications in small molecule and polymer synthesis and has been reviewed [74–76]. The importance of the thiol-ene reaction in the synthesis of β-hydroxy sulfides is exemplified by the work of Scanlan and co-workers as shown in Scheme 41 [77–78]. Treatment of the thiol-ene **113** with the radical initiator 2,2-dimethoxy-2-phenylacetophenone (DPAP) and the photosensitizer 4-methoxyacetophenone (MAP) in DMF ensued to the formation of the thiyl radical **114** that underwent intramolecular cyclization to provide a mixture of biologically important thiosugars **116** and **118** (Scheme 41) [77–78].

### 4. Applications in target-oriented synthesis

**Scheme 42:**
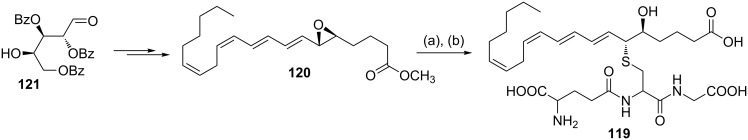
Synthesis of leukotriene C-1 by Corey et al.: (a) *N*-(trifluoroacetyl)glutathione dimethyl ester (3 mol equiv), Et_3_N, MeOH, 23 °C, 4 h, 80%; (b) K_2_CO_3_ (0.1 M) in H_2_O/MeOH (95:5), 23 °C, 12 h.

As indispensable biological agents, leukotrienes have inspired a great deal of clinical research [79], and in support of this, a number of de novo laboratory syntheses have been reported and reviewed [80–81]. In the notable synthesis of leukotriene C-1 (**119**), Corey and co-workers synthesized (−)-methyl *trans*-5(*S*),6(*S*)-oxido-7,9-*trans*-11,14-*cis*-eicosatetraenoate leukotriene A methyl ester (**120**), the key intermediate in its synthesis and biosynthesis, starting from the 2,3,5-tribenzoyl derivative of D-(−)-ribose [82–83]. A single coupling product was obtained from the reaction of *N*-(trifluoroacetyl)glutathione dimethyl ester and triethylamine in methanol at 23 °C for 4 h in 80% yield, which occurred via S_N_2 displacement by nucleophilic sulfur at C-6. Selective hydrolysis of the trifluoroacetyl triester in a mixture of potassium carbonate and potassium bicarbonate in 95:5 water–methanol at 23 °C for 12 h proceeded quantitatively to afford the product leukotriene C-1 (Scheme 42). The epoxide **120** has been the key intermediate in all syntheses of LTC4, -D4, and -E4; upon exposure to the desired amino acid, it yields the required leukotriene [84–98].

**Scheme 43:**
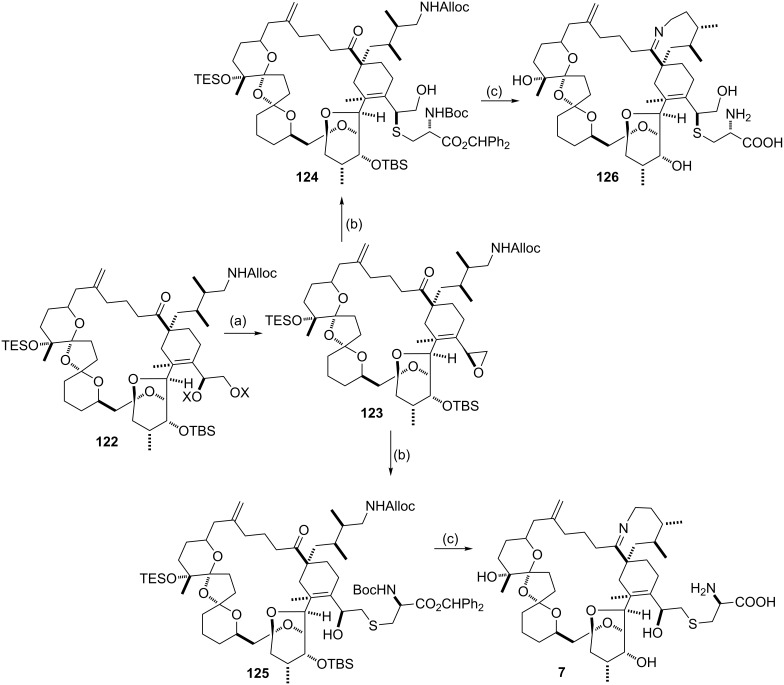
Synthesis of pteriatoxins with epoxide thiolysis to attain β-hydroxy sulfides. Reagents: (a) (1) K_2_CO_3_; (2) HF∙Py, Py, 74% over 2 steps; (2) TsCl; (3) K_2_CO_3,_ 78% over 2 steps; (b) *N*-Boc-L-Cys(SH)-OCHPh_2_, 85%; (c) (1) [Pd(PPh_3_)_4_], AcOH, 72%; (2) 1,3,5-(iPr)_3_C_6_H_2_CO_2_H/Et_3_N salt 80 °C, xylene; (3) TFA, CH_2_Cl_2_, followed by HPLC separation of **7** and **126**.

Epoxide thiolysis to furnish β-hydroxy sulfides was also elegantly utilized by Kishi and co-workers in their landmark synthesis of pteriatoxins and their diastereomers [99]. The advanced intermediate **122** was converted to the epoxide **123**, which upon thiolysis with a strategically protected cysteine, gave the regioisomeric hydroxy sulfides **124** and **125** (Scheme 43). Each of these was then converted to natural pteriatoxin A (**7**) and its analog **126**.

Epoxide opening to yield β-hydroxy sulfides of unnatural (artificial) origin as potential therapeutic agents is also well documented. For instance, Luly et al. synthesized β-hydroxy sulfides coupled to leucine-valine (Leu-Val) replacements as inhibitors of human renin in order to lower blood pressure [100–101].

**Scheme 44:**
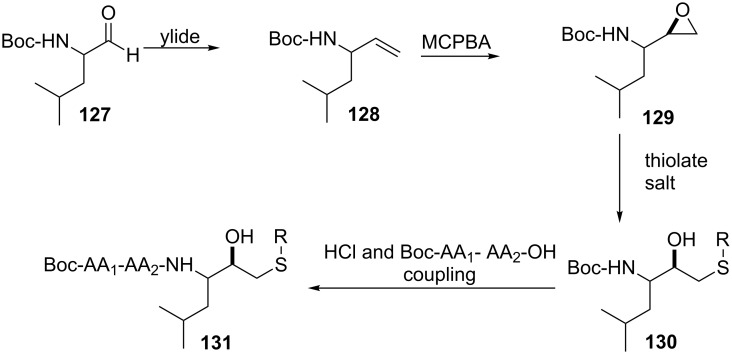
Synthesis of peptides containing a β-hydroxy sulfide moiety.

The high activity of these compounds is attributed to the presence of the hydroxy functional group acting as a transition-state analog. The synthesis of the inhibitors commenced with the conversion of aldehyde **127** into alkene **128** via a Wittig reaction followed by epoxidation to furnish epoxide **129**. Regioselective opening of the epoxide ring with a thiolate gave the *N*-Boc protected β-hydroxy sulfide **130** as a key intermediate. Deprotection of the Boc followed by the imidation of the free amine with protected dipeptides that mimic Leu-Val produced the inhibitors of human renin **131** as depicted in Scheme 44.

**Scheme 45:**
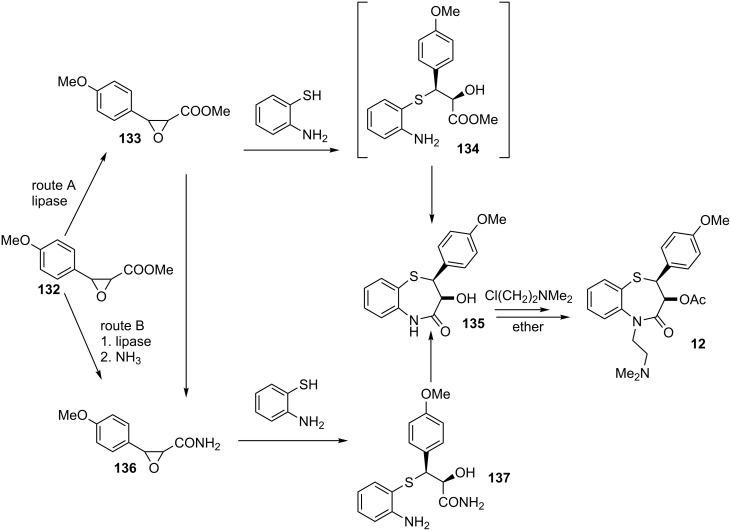
Synthesis of diltiazem (**12**) using biocatalytic resolution of an epoxide followed by thiolysis.

Yoshioka and co-workers reported two synthetic routes for the synthesis of diltiazem (**12**) as depicted in Scheme 45 [102]. The first route (route A) involved the biocatalytic resolution of the racemate epoxide **132** into pure enantiomer **133**, which underwent regio- and stereoselective ring opening using 2-aminobenzenethiol as the nucleophile to afford β-hydroxy sulfide **134** that subsequently underwent intramolecular cyclization into thiazepine **135**. Further manipulations led to the formation of diltiazem (**12**) as its hydrochloride salt. The second route (route B), which is similar to the first, entailed biocatalytic resolution of the enantiomers using a lipase, after which the methyl ester was transformed into a primary amide to provide **136**, which was subsequently subjected to regio- and stereoselective thiolysis with 2-aminobenzenethiol to afford β-hydroxy sulfide **137**. Cyclization of this intermediate led to the formation of **135**. A review of syntheses of the optically active 1,5-benzothiazepines has been done by Lévai and Kiss-Szikszai [103].

## Conclusion

Sulfur, with its ability to exist in various oxidation states and thus various combinations with other atoms, introduces additional intriguing complexity to the molecular architecture of natural products. Many of these sulfur-containing natural products have significant biological activities, which have inspired efforts into their syntheses. The β-hydroxy sulfide moiety is found ubiquitously in natural products and some designer compounds of clinical significance and methods for their preparation have thus for quite a while been an active area of research. Two popular routes to access β-hydroxy sulfides are the thiolysis of epoxides as well as the thiofunctionalization of alkenes, and these have found application in target oriented synthesis. Enantioselective variations of these approaches are also well documented and new ones continue to be investigated. Although a number of methodologies are reported for their synthesis, there is still room for improvement in terms of finding more efficient asymmetric catalysts as well as heterogeneous catalyst systems for the hydroxysulfenylation of alkenes and their respective applications.

## References

[1] Kornprobst J-M, Sallenave C, Barnathan G (1998). Comp Biochem Physiol.

[2] Blunt J W, Carroll A R, Copp B R, Davis R A, Keyzers R A, Prinsep M R (2018). Nat Prod Rep.

[3] Prinsep M R, Rahman A-U (2003). Sulfur-containing natural products from marine invertebrates. Studies in Natural Products Chemistry.

[4] Andersen K K, Bernstien D T, Caret R L, Romanczyk L J (1982). Tetrahedron.

[5] Block E (1992). Angew Chem, Int Ed Engl.

[6] Wright A E, Forleo D A, Gunawardana G P, Gunasekera S P, Koehn F E, McConnell O J (1990). J Org Chem.

[7] Igarashi Y, Asano D, Sawamura M, In Y, Ishida T, Imoto M (2016). Org Lett.

[8] Meng L-H, Wang C-Y, Mándi A, Li X-M, Hu X-Y, Kassack M U, Kurtán T, Wang B-G (2016). Org Lett.

[9] Dunbar K L, Scharf D H, Litomska A, Hertweck C (2017). Chem Rev.

[10] Takada N, Umemura N, Suenaga K, Uemura D (2001). Tetrahedron Lett.

[11] Foster H R, Fuerst E, Branchett W, Lee T H, Cousins D J, Woszczek C (2016). Sci Rep.

[12] Xie Z, Zhou L, Guo L, Yang X, Qu G, Wu C, Zhang S (2016). Org Lett.

[13] de Castro M V, Ióca L P, Williams D E, Costa B Z, Mizuno C M, Santos M F C, de Jesus K, Ferreira É L F, Seleghim M H R, Sette L D (2016). J Nat Prod.

[14] O'Connor S E, Grosset A, Janiak P (1999). Fundam Clin Pharmacol.

[15] Feng J-B, Wu X-F (2016). ChemistryOpen.

[16] Padwa A, Murphree S (2006). ARKIVOC.

[17] Bergmeier S C, Lapinsky J D (2009). Chapter 3: Three-Membered Ring Systems. Progress in Heterocyclic Chemistry.

[18] Bergmeier S C, Lapinsky J D, Gribble G W, Joule G A (2013). Chapter 2: Three-Membered Ring Systems. Progress in Heterocyclic Chemistry.

[19] Page P C B, Wilkes R D, Reynolds D, Katritzky A R, Meth-Cohn, O, Rees C W (1995). Alkyl Chalcogenides: Sulfur-based Functional Groups In Comprehensive Organic Functional Group Transformations.

[20] Posner G H, Rogers D Z (1977). J Am Chem Soc.

[21] Fringuelli F, Pizzo F, Tortoioli S, Vaccaro L (2003). Tetrahedron Lett.

[22] Fan R-H, Hou X-L (2003). J Org Chem.

[23] Fringuelli F, Pizzo F, Tortoioli S, Vaccaro L (2003). J Org Chem.

[24] Caddick S, Fitzmaurice R (2009). Tetrahedron.

[25] Pironti V, Colonna S (2005). Green Chem.

[26] Su W, Chen J, Wu H, Jin C (2007). J Org Chem.

[27] Chen J, Wu H, Jin C, Zhang X, Xie Y, Su W (2006). Green Chem.

[28] Rostami A, Jafari H (2008). S Afr J Chem.

[29] Cossy J, Bellosta V, Hamoir C, Desmurs J-R (2002). Tetrahedron Lett.

[30] Hodgson D M, Gibbs A R, Lee G P (1996). Tetrahedron.

[31] Jacobsen E N (2000). Acc Chem Res.

[32] Pastor I M, Yus M (2005). Curr Org Chem.

[33] Schneider C (2006). Synthesis.

[34] Matsunaga S (2012). Compr Chirality.

[35] Wang P-N (2013). Beilstein J Org Chem.

[36] Borissov A, Davies T Q, Ellis S R, Fleming T A, Richardson M S W, Dixon D J (2016). Chem Soc Rev.

[37] Yamashita H, Mukaiyama T (1985). Chem Lett.

[38] Iida T, Yamamoto N, Sasai H, Shibasaki M (1997). J Am Chem Soc.

[39] Wu M H, Jacobsen E N (1998). J Org Chem.

[40] Wu J, Hou X-L, Dai L-X, Xia L-J, Tang M-H (1998). Tetrahedron: Asymmetry.

[41] Zhou Z, Li Z, Quanyong W, Liu B, Li K, Zhao G, Zhou Q, Tang C (2006). J Organomet Chem.

[42] Sun J, Yuan F, Yang M, Pan Y, Zhu C (2009). Tetrahedron Lett.

[43] Sun J, Yang M, Yuan F, Jia X, Yang X, Pan Y, Zhu C (2009). Adv Synth Catal.

[44] Boudou M, Ogawa C, Kobayashi S (2006). Adv Synth Catal.

[45] Ogawa C, Kobayashi S, Ojima I (2010). Catalytic Asymmetric Synthesis in Nonconventional Media/Conditions. Catalytic Asymmetric Synthesis.

[46] Kitanosono T, Masuda K, Xu P, Kobayashi S (2018). Chem Rev.

[47] Nandakumar M V, Tschop A, Krautscheid H, Schneider C (2007). Chem Commun.

[48] Tschöp A, Nandakumar M V, Pavlyuk O, Schneider C (2008). Tetrahedron Lett.

[49] Nandakumar M V, Ghosh S, Schneider C (2009). Eur J Org Chem.

[50] Chen Y-J, Chen C (2007). Tetrahedron: Asymmetry.

[51] Wang Z, Law W K, Sun J (2013). Org Lett.

[52] Ingle G, Mormino M G, Antilla J C (2014). Org Lett.

[53] Kumar G, Kumar G, Gupta R (2016). RSC Adv.

[54] Cho B T, Choi O K, Kim D J (2002). Tetrahedron: Asymmetry.

[55] Cho B T, Shin S H (2005). Tetrahedron.

[56] Arroyo Y, Rodríguez J F, Santos M, Tejedor M A S, Ruano J L G (2007). J Org Chem.

[57] Fujisawa T, Itoh T, Sato T (1984). Tetrahedron Lett.

[58] Kutney G W, Turnbull K (1984). J Chem Educ.

[59] Soleiman-Beigi S, Kohzadi H (2014). Arabian J Chem.

[60] Devan N, Sridhar P R, Prabhu K R, Chandrasekaran S (2002). J Org Chem.

[61] Yu H, Dong D, Ouyang Y, Wang Y, Liu Q (2007). Synlett.

[62] Zhu J, Li R, Ge Z, Cheng T, Li R (2009). Chin J Chem.

[63] Surendra K, Krishnaveni N S, Sridhar R, Rao K R (2006). J Org Chem.

[64] Deraedt C, Astruc D (2016). Coord Chem Rev.

[65] Movassagh B, Navidi M (2008). Tetrahedron Lett.

[66] Zhou S-F, Pan X, Zhou Z-H, Shoberu A, Zou J P (2015). J Org Chem.

[67] Yadav V K, Srivastava V P, Yadav L D S (2015). Tetrahedron Lett.

[68] Bewick A, Mellor J M, Milano D, Owton W M (1985). J Chem Soc, Perkin Trans 1.

[69] Taniguchi N (2006). J Org Chem.

[70] Yadav L D, Awasthi C (2009). Tetrahedron Lett.

[71] Muangkaew C, Katrun P, Kanchanarugee P, Pohmakotr M, Reutrakul V, Soorukram D, Jaipetch T, Kuhakarn C (2013). Tetrahedron.

[72] Marakalala M B, Kinfe H H (2017). Eur J Org Chem.

[73] Denmark S E, Kornfilt D J P, Vogler T (2011). J Am Chem Soc.

[74] Hoyle C E, Lowe A B, Bowman C N (2010). Chem Soc Rev.

[75] Hoyle C E, Bowman C N (2010). Angew Chem, Int Ed.

[76] Scanlan E M, Corcé V, Malone A (2014). Molecules.

[77] Malone A, Scanlan E M (2013). Org Lett.

[78] Malone A, Scanlan E M (2013). J Org Chem.

[79] Grice C A, Fourie A M, Lee-Dutra A, Levin J I, Laufer S (2012). Leukotriene A4 Hydrolase: Biology, Inhibitors and Clinical Applications. Anti-Inflammatory Drug Discovery.

[80] Ackroyd J, Scheinmann F (1982). Chem Soc Rev.

[81] Lai S M F, Manley P W (1984). Nat Prod Rep.

[82] Corey E J, Clark D A, Goto G, Marfat A, Mioskowski C, Samuelsson B, Hammarstroem S (1980). J Am Chem Soc.

[83] Corey E J, Arai Y, Mioskowski C (1979). J Am Chem Soc.

[84] Gleason J G, Bryan D B, Kinzig C M (1980). Tetrahedron Lett.

[85] Rokach J, Girard Y, Guindon Y, Atkinson J G, Larue M, Young R N, Masson P, Holme G (1980). Tetrahedron Lett.

[86] Rosenberger M, Neukom C (1980). J Am Chem Soc.

[87] Baker S R, Jamieson W B, McKay S W, Morgan S E, Rackham D M, Ross W J, Shrubsall P R (1980). Tetrahedron Lett.

[88] Rokach J, Young R N, Kakushima M, Lau C-K, Seguin R, Frenette R, Guindon Y (1981). Tetrahedron Lett.

[89] Rokach J, Zamboni R, Lau C-K, Guindon Y (1981). Tetrahedron Lett.

[90] Ernest I, Main A J, Menassé R (1982). Tetrahedron Lett.

[91] Cohen N, Banner B L, Lopresti R J (1980). Tetrahedron Lett.

[92] Rokach J, Lau C-K, Zamboni R, Guindon Y (1981). Tetrahedron Lett.

[93] Marriot D P, Bantick J R (1981). Tetrahedron Lett.

[94] Weller P F, Lee C W, Foster D W, Corey E J, Austen K F, Lewis R A (1983). Proc Natl Acad Sci U S A.

[95] Atrache V, Pai J-K, Sok D-E, Sih C J (1981). Tetrahedron Lett.

[96] Rosenberger M, Newkom C, Aig E R (1983). J Am Chem Soc.

[97] Delorme D, Girard Y, Rokach J (1989). J Org Chem.

[98] Klotz P, Foucaud B, Goeldner M P, Hirth C G (1993). J Org Chem.

[99] Matsuura F, Peters R, Anada M, Harried S S, Hao J, Kishi Y (2006). J Am Chem Soc.

[100] Luly J R, Yi N, Soderquist J, Stein H, Cohen J, Perun T J, Plattner J J (1987). J Med Chem.

[101] Webb R L, Schiering N, Sedrani R, Maibaum J (2010). J Med Chem.

[102] Yamada S-i, Tsujioka I, Shibatani T, Yoshioka R (1999). Chem Pharm Bull.

[103] Lévai A, Kiss-Szikszai A (2008). ARKIVOC.

